# Halogen bonding and host–guest chemistry between *N*-alkylammonium resorcinarene halides, diiodoperfluorobutane and neutral guests

**DOI:** 10.3762/bjoc.15.91

**Published:** 2019-04-18

**Authors:** Fangfang Pan, Mohadeseh Dashti, Michael R Reynolds, Kari Rissanen, John F Trant, Ngong Kodiah Beyeh

**Affiliations:** 1 College of Chemistry, Key Laboratory of Pesticide & Chemical Biology of Ministry of Education, Hubei International Scientific and Technological Cooperation Base of Pesticide and Green Synthesis, Central China Normal University, Luoyu Road 152, Wuhan, Hubei Province, 430079, People's Republic of China; 2 University of Windsor, Department of Chemistry and Biochemistry, Windsor, Ontario, 401 Sunset Avenue, N9B 3P4, Canada; 3 University of Jyvaskyla, Department of Chemistry, PO Box 35, Jyväskylä, FIN-40014, Finland; 4 Oakland University, Department of Chemistry, 146 Library Drive, Rochester, Michigan, 48309-4479, USA

**Keywords:** capsule, dimeric assemblies, halogen bonding, host–guest chemistry, resorcinarene salts, X-ray crystallography

## Abstract

Single crystal X-ray structures of halogen-bonded assemblies formed between host *N*-hexylammonium resorcinarene bromide (**1**) or *N*-cyclohexylammonium resorcinarene chloride (**2**), and 1,4-diiodooctafluorobutane and accompanying small solvent guests (methanol, acetonitrile and water) are presented. The guests’ inclusion affects the geometry of the cavity of the receptors **1** and **2**, while the divalent halogen bond donor 1,4-diiodooctafluorobutane determines the overall nature of the halogen bond assembly. The crystal lattice of **1** contains two structurally different dimeric assemblies A and B, formally resulting in the mixture of a capsular dimer and a dimeric pseudo-capsule. ^1^H and ^19^F NMR analyses supports the existence of these halogen-bonded complexes and enhanced guest inclusion in solution.

## Introduction

The construction of specific supramolecular assemblies based on the directional non-covalent bonding has been a central goal of supramolecular chemistry and materials science [1–3]. New systems both help us to better understand the nature and impetus behind the self-assembly of these fascinating systems, while also providing new materials that can provide the basis for a wide number of applications [4–5]. Halogen bonding (XB), as a type of directional non-covalent interaction, is regarded as the “long lost brother” of hydrogen bonding (HB) [6–7]. Although XB in many aspects is very similar to HB, and in most cases not as strong as classical HB, the character of XB, such as hydrophobicity, adjustability, or softness, allows these interactions to be used in aqueous or polar environments where HB-based systems are less viable [8–12]. However, we lack the analytical tools to directly examine and determine the precise structure of XB assemblies in solution [13–15]. In the solid-state, X-ray diffraction has proven an incredibly effective tool for observing privileged conformations and structures [15–19]. From the crystallographic information we can extract the polymorphism, high Z’-value, twining, and disorder that provide insight into the dynamics of prenucleation assembly, nucleation, and crystal growth, which, in turn, provide information regarding the nature of assemblies in solution [20–25]. In particular, a high Z’-value structure is sometimes regarded as a “fossil” of the solute in solution, since the symmetry independent molecules have a great deal of influence on the way in which the crystals form [26]. Twinning and disorder are generally seen as an undesirable complication in determining structures; however, the nature and extent of the disorder contains significant information regarding the dynamics and conformational sampling of the molecules [19,24,27]. Desiraju et al. employing substitutional disorder, achieved the co-crystallization of six components [28]. As for positional disorder, it generally indicates the same molecule or assembly can adopt more than one favourable conformation. From this perspective, disorder can be considered as a special case of a co-crystal or high Z’-value structure. Disorder is not inherently a feature of a poor structure, disorder instead indicates the complexity of the dynamic solution state. Solving it, however, remains a challenge.

*N*-Alkylammonium resorcinarene halides (NARXs) have been extensively studied in our groups as multidentate halogen bond acceptors [29–34]. We have previously shown that *N*-alkylammonium resorcinarene bromides (NARBrs) can form various halogen-bonded assemblies with the classical organic halogen bond donor 1,4-diiodooctafluorobutane (DIOFB) depending on the solvent, the presence of potential guests, and the length of the alkyl chain [30–31]. In our previous report, the basic conformation of the host *N*-hexylammonium resorcinarene bromide (Hex-NARBr) was driven by the incorporation of a 1,4-dioxane guest molecule [32], and the inter-cavitand bridging of DIOFB. The relatively long *N*-hexylammonium groups endow the constructs with significant flexibility. Thus, different structures of the same XB acceptor–donor pair could be obtained by simply changing the solvent. The solvent does not appear to be critical to the halogen bonded assembly, and this raised the question as to whether crystals arising from solvent mixtures could be useful in probing the solvation interactions in solution.

**Figure 1:**
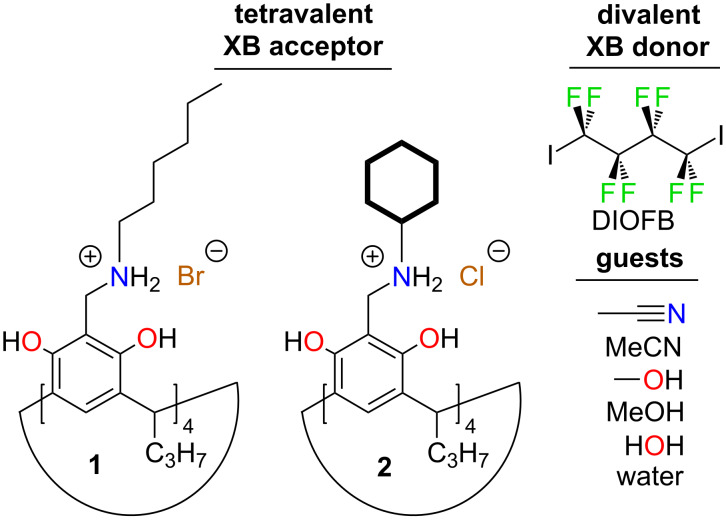
Tetravalent XB acceptor, Hex-NARBr **1**, Cy-NARCl **2**, divalent XB donor DIOFB, and guests MeCN, MeOH and water.

In the current study, we examine the role of the inclusion guest in determining the final XB structures. Instead of adding 1,4-dioxane as a guest, we use methanol and acetonitrile as both solvent and as potential inclusion guests (Figure 1). The flexibility imparted to the host due to the lack of a defined inclusion guest in these systems is considerable and led to many amorphous systems, as expected. However, two samples were successfully crystallized and structurally characterized by single X-ray crystallography: MeOH-MeCN@**1**&DIOFB and Water@**2**&DIOFB. These two structures, besides illustrating the potential of halogen bonding for organizing complex capsular systems, shed light on the importance of flexibility in affecting the self-assembled systems.

## Results and Discussion

### Single crystal X-ray diffraction

**Figure 2:**
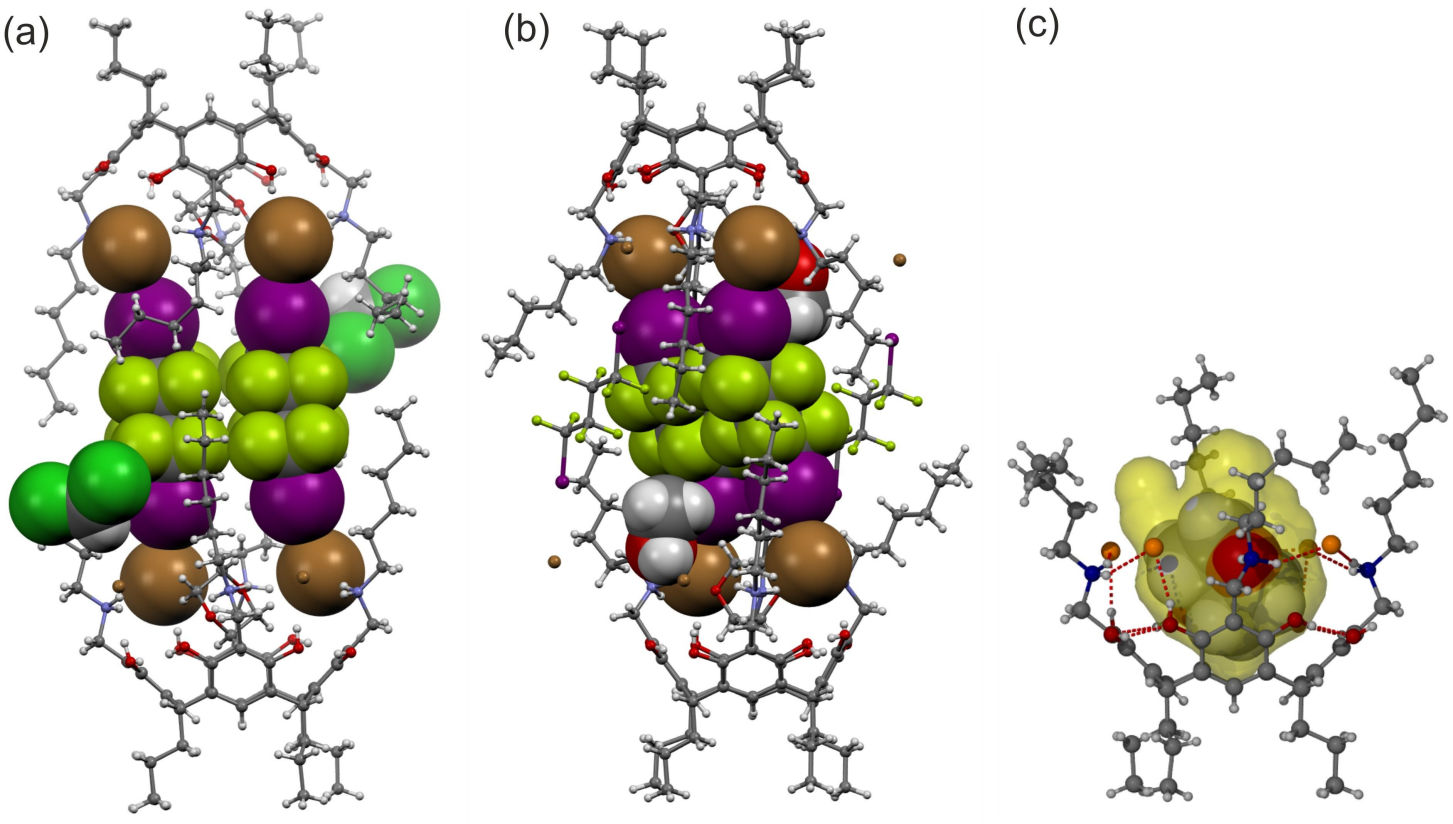
The previously reported halogen-bonded complexes CHCl_3_@**1**&DIOFB (a), and MeOH@**1**&DIOFB (b). (c)The fit of 1,4-dioxane within the cavity of **1** [32].

The *endo*-inclusion of guests by NARXs greatly influences the geometry of the system and directly affects the orientation of the upper rim arms. This is particularly true for the NARX derivatives with long chain upper rim substituents. We have previously observed that even with the most suitable inclusion guest (1,4-dioxane), the lattice solvent molecules, regardless of their polarity, insert between two *N*-hexyl arms using an OH/CH···Br^−^ hydrogen bond (Figure 2) [32]. To better understand whether this is fundamental to these systems, or a result of the enforced geometry caused by the included guest, we have extended this study in this current report by excluding the 1,4-dioxane molecule as obvious inclusion guests.

**Figure 3:**
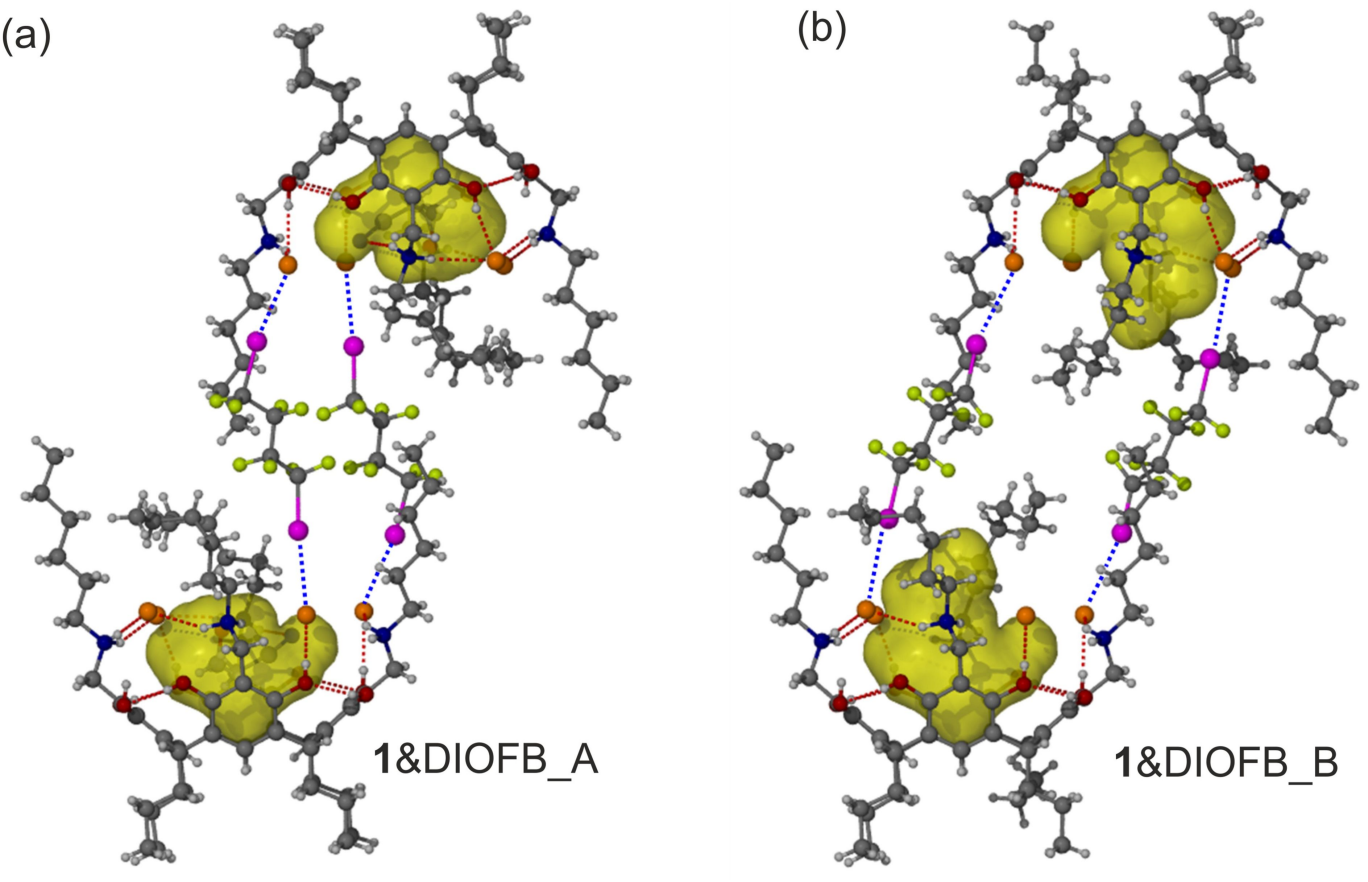
The two dimerization modes in the MeOH-MeCN@**1**&DIOFB complex. In both modes, the cavities are shown as transparent yellow cloud.

The newly isolated crystal, MeOH-MeCN@**1**&DIOFB, is formed as a 2:2 (**1**:DIOFB) halogen-bonded species encapsulating approximately equimolar amounts of MeCN and MeOH in the cavity of the resorcinarenes. Significant disorder is present in this cavity, viz, half MeCN and half MeOH were assigned as disorder in the cavity of **1**. According to the previously reported structure, when 1,4-dioxane was the inclusion guest, the volume of the cavity is above 170 Å^3^, much larger than that of MeCN and MeOH, which are ca. 41 and 53 Å^3^, respectively [35]. Hence, the resorcinarene has to deform to adapt to the small guests by maximizing the contacts between the host and guest (Figure 3). In detail, in the MeCN occupied systems, the N atom is stabilized by an NH···N hydrogen bond and a weak pnictogen bond to a Br^−^ counterion with an *R*_NB_ of 0.96 (Supporting Information File 1, Figure S2). MeOH-stabilized systems instead employ weak NH···O and OH···N hydrogen bonds (Supporting Information File 1, Figure S2). In both cases, the electron-rich environment of the cavity provides a negative electrostatic surface for interactions with the electropositive methyl groups of either MeCN or MeOH causing the orientation to be similar in both cases. Deformation of the resorcinarene is a result of the small solvent size leading to a decrease in internal cavity volume of the receptor. The calculated space sizes are 79.45 Å^3^ in MeOH@**1** and 101.17 Å^3^ in MeCN@**1** (*r*_probe_ = 1.2 Å) [34]. The deformation also shifts the relative positions of the *N*-alkyl “arms” and the halide anions, which additionally change the relative orientation of DIOFB XB donors when directional halogen bonding forms. Two DIOFB linked two MeOH-MeCN@**1** complexes, similar to that observed with our previous chloroform-involved 2:2 halogen-bonded complex [31]. In the present assembly, no solvent molecules are found in the encapsulated volume outlined by the two DIOFB molecules and so the two cavitands could be assigned. The absence of the guest molecules, and the resulting empty space, induces disorder for both the hexyl groups on the upper-rim of the cavitand, and the DIOFB molecules. Two preferred conformations of these systems were identified with a ratio of ca. 3:1 (Figure 3, Figures S1 and S3 in Supporting Information File 1). Due to the center of inversion, the two conformations show different dimerization modes (Supporting Information File 1, Figure S3). In **1**&DIOFB_A, the two hex-NARBrs are linked by two DIOFB molecules via four Br···I halogen bonds with average *R*_XB_ of 0.86. The two participating bromide anions are on adjacent “arms”; the remaining two Br^−^ counterions are covered by the bent hexyl “arms”. In **1**&DIOFB_B, the two DIOFB molecules link the two hex-NARBrs using the diametrically opposed Br^−^ anions. The average *R*_XB_ of these XBs is 0.98. In this second conformer, a space was created between the hosts, thus the hexyl groups bend inwards to fill the space. The relatively strong halogen bonds in **1**&DIOFB_A partially account for its larger population occupancy than **1**&DIOFB_B. In both modes, there is no solvent-accessible space between the dimerized resorcinarene salts. The halogen bond donors connect the two resorcinarene like a solid tube, only creating isolated pores in the cavity of the resorcinarene. Note that the two modes are present simultaneously in the crystal lattice, thus, the structure formally contains around 75% halogen bonded dimer, and 25% halogen bonded capsule. Additionally, the conformation difference could reveal the motion of the molecules in solution, and the evaporation of the solvent molecules during crystal growth. Waving of the hexyl groups also affects one of the propyl groups in the lower rim of the resorcinarene. As a result, the two conformers A and B also differentiate from each other in the lower rim.

**Figure 4:**
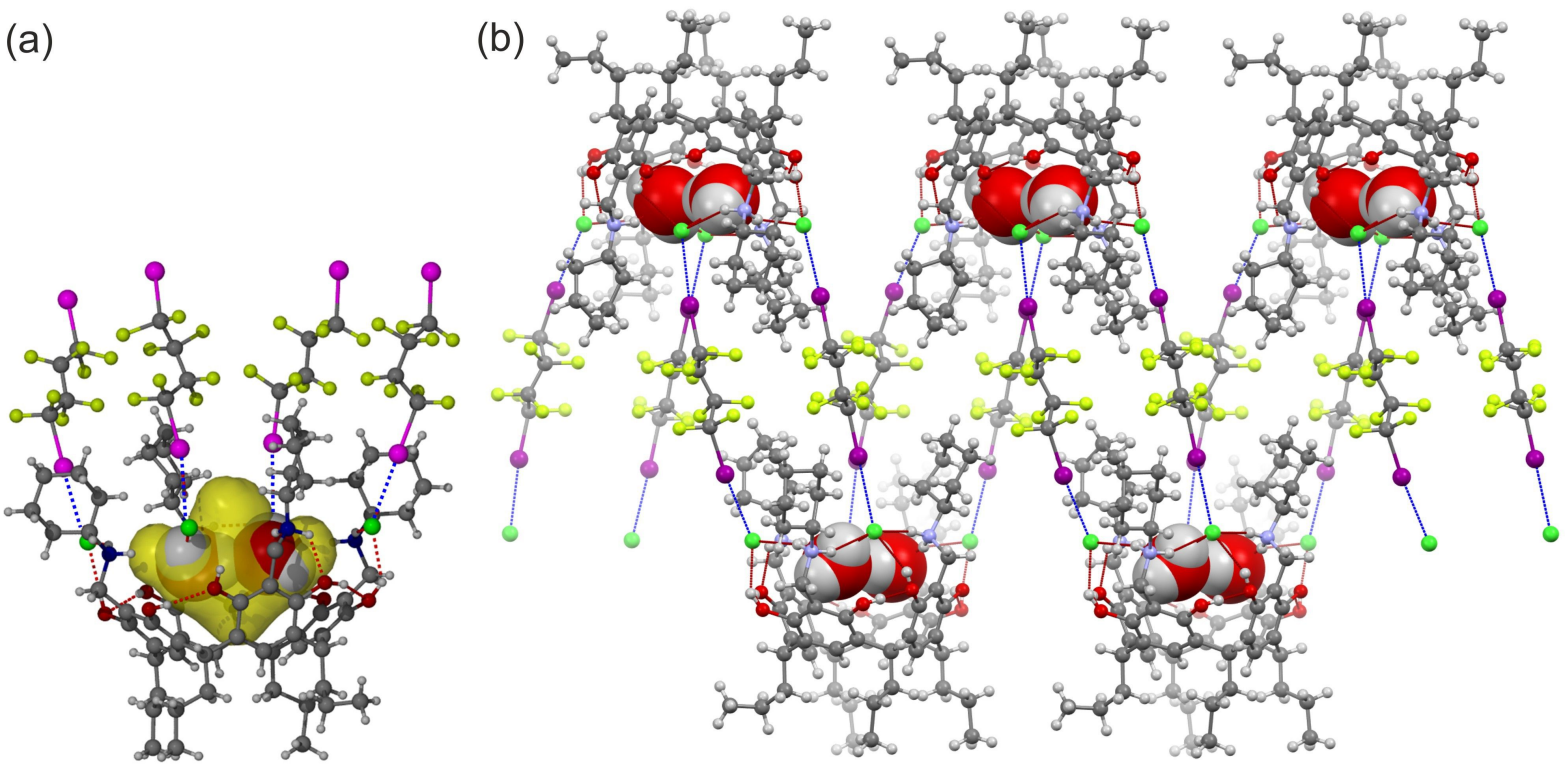
(a) The halogen bonding (blue broken lines), hydrogen bonding (red broken lines) and the host–guest effect (water molecules in CPK, and the cavity space in transparent yellow cloud) in Water@**2**&DIOFB; (b) The halogen bonded polymer. Water molecules in CPK mode and all the other in ball-and-stick type.

A similar study was also extended to another resorcinarene chloride salt, Cy-NARCl **2**. The obtained crystal showed the assembly as Water@**2**&DIOFB, forming a 1:2 (**2**:DIOFB) halogen bonded chain (Figure 4). In this case, the cavitand encapsulated two water molecules in the cavity. The water molecules were positioned at the same level as the (HNH···Cl^−^)_4_ HB circle. The O···Cl^−^ distances of 3.13(3) and 3.22(4) Å suggest OH···Cl^−^ hydrogen bonds. Meanwhile, an OH···O hydrogen bond should also exist between the two water molecules with the O···O distance of 3.01(5) Å. It is surprising to find that the resorcinarene cavity below these water molecules is empty. It seems the electronegative surface of the cavity repels the water molecules and pushes them up (Figure 4). Besides the hydrogen bonds, each Cl^−^ anion also donates electron density to one DIOFB molecule via a halogen bond with an average *R*_XB_ of 0.88. The four DIOFB molecules bound with **2** can be classified into two groups by virtue of their directionality. Each group links to another resorcinarene chloride salt through an XB. The XB interactions consequently organize the cavitands along the crystallographic *c* axis. This binding mode is different from all the other reported halogen bonded assemblies observed for NARXs. We account for the staggered connection in Water@**2**&DIOFB by the twist of the resorcinarene framework that results in the absence of a geometry-supporting inclusion guest. The presence of the halogen bond donors covers the cavity of the resorcinarene and creates a pore with volume of ca. 114.51 Å^3^ (*r*_probe_ = 1.2 Å). The two encapsulated water molecules only take up 33.9% of this pore, much lower than the 55% that would be expected based on Rebek’s rule [36].

### NMR spectroscopy

Solvent interference is a key limiting factor in observing halogen bonds in solution. Despite this limitation, NMR spectroscopy is one of the most powerful tools for observing and studying XB systems in solution [37–41]. The bromide anions of the NARBrs are hydrogen-bonded to the hydrogens of the ammonium groups and to the phenolic hydroxy groups. Halides such as chloride and bromides can have high coordination numbers and as such can simultaneously be involved in both HB and XB to form ordered assemblies [32–33]. When the already hydrogen-bonded halides in NARXs are involved in XB, changes in the ^1^H NMR chemical shifts of the –OH and –NH_2_ protons of the NARXs are expected [32–33]. Additionally, NARXs are also known to cooperatively bind small guest molecules such as mono- and diamides [42–43]. Consequently, we used ^1^H and ^19^F NMR spectroscopy to study the XB assemblies formed between Hex-NARBr **1** and 1,4-diiodooctafluorobutane (**5**) in the presence of the solvent guests (MeOH and MeCN) in chloroform. For this study, we prepared samples including the pure components as well as three experimental mixtures: a 1:2 mixture of *N*-hexyl NARBr **1** and XB donor DIOFB; and a 1:2:2 mixture of host, XB-donor, and either methanol or acetonitrile. The ^1^H and ^19^F NMR spectra of all these samples were recorded at 298 K and analyzed.

**Figure 5:**
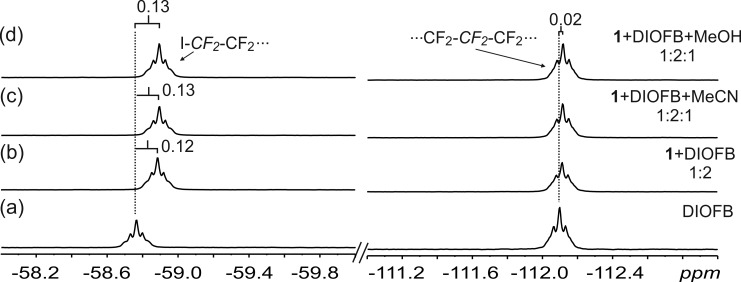
^19^F NMR in CDCl_3_ at 298 K of: a) DIOFB (10 mM); b) 1:2 mixture of DIOFB and **1**; 1:2:1 mixture of DIOFB, **1**, and c) MeCN, d) MeOH. The dashed lines give an indication of the signal changes in ppm, resulting from the formation of XBs. The dashed lines give an indication of the signal changes in ppm.

In the ^19^F NMR analyses, the fluorine signals of the XB donor DIOFB were monitored. In all cases, minor upfield shifts of the fluorines on the terminal carbons were observed (0.12 ppm in **1**·DIOFB, 0.13 ppm in **1**·DIOFB·MeOH and **1·**DIOFB·MeCN, Figure 5). Minimal changes (<0.03 ppm) were observed for the fluorine atoms attached to the internal carbon atoms. This strongly suggests that a similar XB involving the iodine atoms of DIOFB exists in solution both with and without the guests (Figure 5).

**Figure 6:**
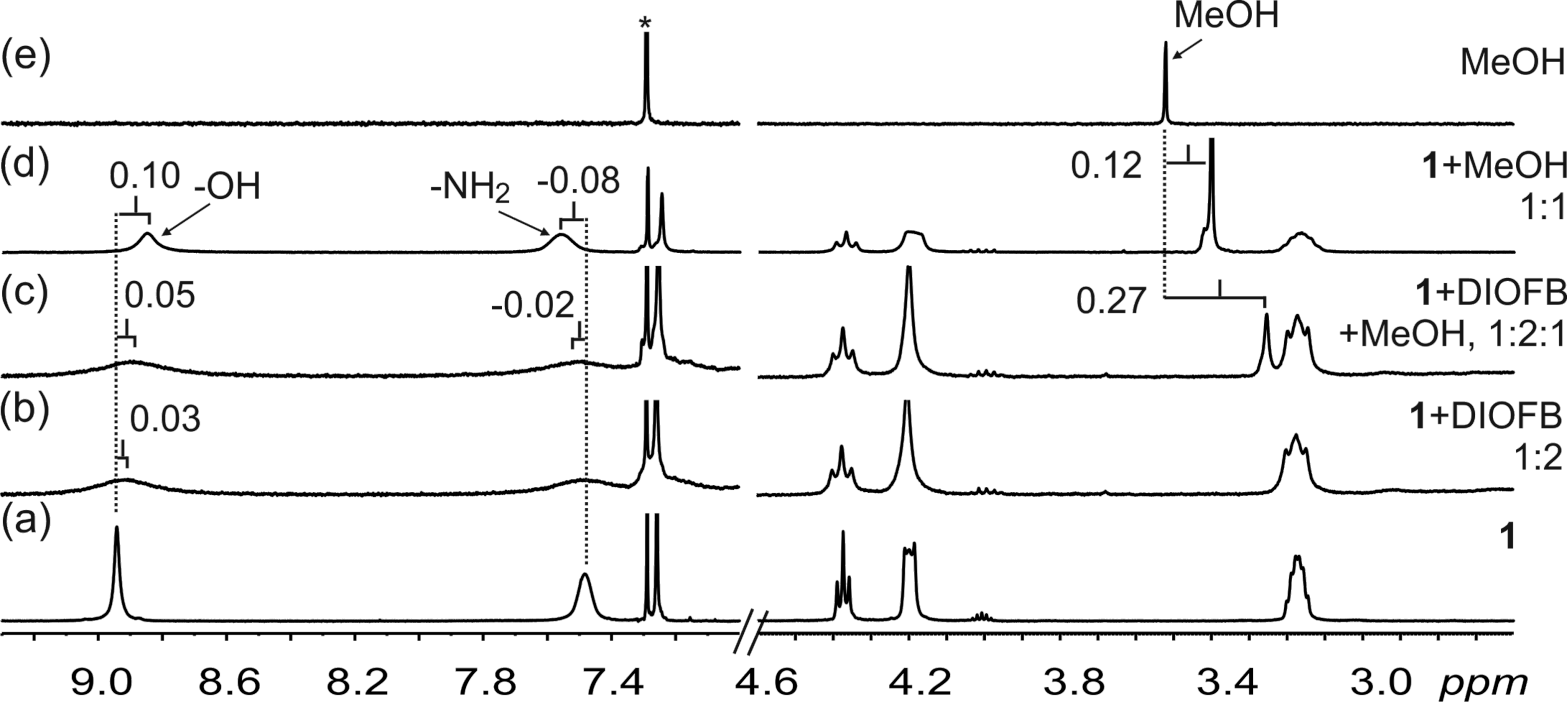
^1^H NMR in CDCl_3_ at 298 K of: a) **1** (10 mM), b) 1:2 mixture of **1** and DIOFB, c) 1:2:1 mixture of **1**, DIOFB and MeOH, d) 1:1 mixture of **1** and MeOH, and e) MeOH (10 mM). The dashed lines give an indication of the signal changes in ppm. The asterisk represents the residual CDCl_3_ solvent.

To complement the direct evidence from ^19^F NMR, ^1^H NMR showed small changes of the –OH and –NH_2_ signals of **1**, attributed to the formation of HB and XB in solution (Figure 5 and Figure S4 in Supporting Information File 1). In the presence of the guests (MeOH and MeCN), ^1^H NMR reveal significant complexation-induced shielding of the guest protons from samples **1**·DIOFB·MeOH and **1**·DIOFB·MeCN. There was a significant increase in the shielding of the guest signals when compared to the host:guest mixtures (**1**·MeOH and **1**·MeCN). Taking the MeOH guest as an example, in the presence of the XB donor DIOFB, the methyl protons of MeOH move 0.27 ppm downfield compared to a more limited 0.12 ppm shift in the absence of the XB donor DIOFB (Figure 6). In the halogen bonded assembly between DIOFB and **1**, the DIOFB blocks the “side windows” of the NARX thus increasing the depth of the cavity. As such the bound guest suffers an increase in the anisotropy influence from the host’s aromatic rings, increasing the shielding.

## Conclusion

In conclusion, we present XB assemblies between Hex-NARBr and Cy-NARCl as tetravalent XB acceptors, a divalent XB donor DIOFB, and small organic guest solvents (MeOH, MeCN and water). In the assemblies, both XB and HB are working in tandem and concertedly to form networks of non-covalent interactions stabilizing the dimeric and capsular structures in the solid state. The inclusion guests affect the geometry of the cavity of the hex-NARBr and cy-NARCl, thus affecting the halogen bonding connection in the final assemblies. In the complex MeOH-MeCN@**1**&DIOFB, because of the crystallographic disorder, two halogen bonded dimerization modes were found in the crystal lattice. In the complex of Water@**2**&DIOFB, two water molecules act as inclusion guests, taking up only 33.9% of the cavity, thus the squeezed resorcinarene chloride prefers to be polymerized via halogen bonds with DIOFB. The ^1^H NMR studies in chloroform for Hex-NARBr and DIOFB clearly confirm the existence of XB in solution through reasonable shift changes of the ^19^F signals of –CF_2_I and small chemical shift changes of the –OH and –NH_2_ signals. Guest binding was confirmed from the increase shielding of the guest signals. These results adds to the literature of small organic guest compounds bound by *N*-alkylammonium resorcinarene halide receptors synergistically via HB and XB interactions.

## Supporting Information

File 1 Experimental details, ^1^H and ^19^F NMR solution data and X-ray crystallographic details.
