# Supplementary material for: Halogen bonding and host–guest chemistry between N-alkylammonium resorcinarene halides, diiodoperfluorobutane and neutral guests
Source: Beilstein J Org Chem. 2019 Apr 18;15:947–54. doi: 10.3762/bjoc.15.91 (PMC6541336; doi:10.3762/bjoc.15.91)
Supplement: File 1 — Experimental details, 1H and 19F NMR solution data and X-ray crystallographic details. [file Beilstein_J_Org_Chem-15-947-s001.pdf]

BEILSTEIN JOURNAL OF ORGANIC CHEMISTRY

Supporting Information

for

Halogen bonding and host–guest chemistry between *N*-alkylammonium resorcinarene halides, diiodoperfluorobutane and neutral guests

Fangfang Pan, Mohadeseh Dashti, Michael R. Reynolds, Kari Rissanen, John F. Trant and Ngong Kodiah Beyeh

Beilstein J. Org. Chem. **2019,** *15,* 947–954. doi:10.3762/bjoc.15.91

Experimental details, ^{1}H and ^{19}F NMR solution data and X-ray crystallographic details

License and Terms: This is a supporting information file under the terms of the Creative Commons Attribution License (http://creativecommons.org/licenses/by/4.0). Please note that the reuse, redistribution and reproduction in particular requires that the authors and source are credited.
The license is subject to the *Beilstein Journal of Organic Chemistry* terms and conditions: (https://www.beilstein-journals.org/bjoc)

I. General information……………………………………………………………………………………….S2
II. Solid state analyses …………………………………………………………………………………..S2
III. NMR Spectroscopy………………………………………………………………………………………..S5
IV. References………………………………………………………………………………………………..S6

I GENERAL INFORMATION

All the solvents used for syntheses and crystal growth were reagent grade and were used as received and were purchased from Sigma-Aldrich or Oakwood Chemicals (Estill SC, USA). The *N*-alkylammonium resorcinarene halides **1** and **2** were synthesized according to reported procedures [1,2]. Briefly, the Mannich [3] condensation reaction between C-$_{propyl}$-resorcinarene and either hexylamine or cylcohexylamine in the presence of excess formaldehyde yields the tetrabenzoxazines. The tetrabenzoxazine ring was opened in the presence of either hydrobromic (*N*-hexyltetrabenzoxazine) or hydrochloric (*N*-cyclohexyltetrabenzoxazine) acid under refluxing conditions to give the *N*-hexylammonium resorcinarene bromide **1** and the *N*-cyclohexylammonium resorcinarene chloride **2**.

II SOLID STATE ANALYSES

X-ray crystallography

Crystals of the assembly MeOH-MeCN@**1**&DIOFB were obtained by slow evaporation of MeOH/MeCN solution containing 1:1 mixture of *N*-hexylammonium resorcinarene bromide **1** and DIOFB. Water@**2**&DIOFB was crystallized from the slow evaporation of wet MeOH solution containing 1:2 mixture of *N*-cyclohexylammonium resorcinarene chloride **2** and DIOFB. All crystallographic data were collected at 123 K with an Agilent Super-Nova diffractometer using mirror-monochromatized Cu-Kα (λ = 1.54184Å) radiation. *CrysAlisPro* [4] was used for both data collection and processing. The intensities were corrected for absorption using the analytical face index absorption correction method [5] for MeOH-MeCN@**1**&DIOFB and a gaussian integration method[4] for Water@**2**&DIOFB. The structures were solved by direct method with *SHELXT* [6] and refined by full-matrix least-squares methods using *OLEX2* [7], which utilizes the *SHELXL-2015* module [8]. All non-hydrogen atoms in the structures were refined with anisotropic thermal parameters, except for some carbon atoms in the disordered hexyl group in MeOH-MeCN@**1**&DIOFB.

In MeOH-MeCN@**1**&DIOFB, one of the four hexyl groups was disordered over three sites with occupancies of 0.375, 0.375 and 0.25, respectively. The two populations with 0.375 occupancy were close to each other, thus it is hard to exactly locate the atoms. Thus “DFIX” command was used to restrain the geometry, and the isotropic thermal vibration parameters of the carbon atoms were kept the same and refined together. The disorder of the hexyl groups also resulted in the disorder distribution of DIOFB molecule and one of the bromide anion. The occupancy of the two parts were 0.75 and 0.25, respectively. Geometry and ADP were also strongly restrained for DIOFB with “DFIX”, “SADI”, “SIMU” and “ISOR”, or constrained with “EADP” command. The inclusion guest was assigned as 50% MeOH and 50% MeCN according to the difference Fourier map. All the hydrogen atoms were introduced at ideal positions with riding mode.

In Water@**2**&DIOFB, two of the cyclohexyl groups and the two DIOFB molecules were disordered. For the cyclohexyl groups, no split was made, however, the geometry was constrained with "DFIX 1.52" and "DANG 2.54" commands, and the anisotropic displacement parameters were restraint with "ISOR 0.02 0.04" and/or "SIMU" commands. The DIOFB molecules were also not split with the exception of the iodine atoms. These were refined with very strong constraints and restraints. The iodine atoms were split over two or three sites according to the differences in the Fourier map. The crystal was twinned with the refined BASF value of "0.49(2)". All the hydrogen atoms bound to carbon and nitrogen were introduced at ideal positions with riding mode. The H atoms bound with oxygen were fixed by "AFIX 3" to the positions that could form suitable hydrogen bonds.

Crystal data MeOH-MeCN@**1**&DIOFB: 0.07×0.17×0.27 mm, $C_{74.5}H_{115.5}N_{4.5}O_{8.5}Br_4F_8I_2$, M = 1923.64, triclinic, space group P-1, a = 12.5314 (6) Å, b = 18.1442(9) Å, c = 21.4895(9) Å, α = 66.772(5)°, β = 79.113(4)°, γ = 70.798(5)°, V = 4230.4(4) Å^3, Z = 2, ρ = 1.510 g cm^{-3}, μ = 8.634 mm^{-1}, F(000) = 1948, 25887 reflections (ϑ_{max} = 76.777°) measured (16892 unique, R_{int} = 0.0373, completeness = 99.1%), Final R indices ($I > 2\sigma(I)$): R_1= 0.0603, wR_2 = 0.1725, R indices (all data): R_1= 0.0761, wR_2 = 0.1725. GOF = 1.060 for 1123 parameters and 173 restraints, largest diff. peak and hole 1.437/–1.107 eÅ^{-3}. CCDC-1894766 contains the supplementary data for this structure.

Crystal data Water@**2**&DIOFB: 0.01×0.14×0.28 mm, $C_{76}H_{108}Cl_4F_{16}I_4N_4O_{10}$, M = 2191.06, orthorhombic, space group $Fdd2$, a = 62.475(2) Å, b = 41.0603(13) Å, c = 14.4679(6) Å, V = 37114(2) Å^3, Z = 16, ρ = 1.569 g cm^{-3}, μ = 12.345 mm^{-1}, F(000) = 17536, 18233 reflections (ϑ_{max} = 66.750°) measured (12019 unique, R_{int} = 0.0791, completeness = 97.9%), Final R indices ($I > 2\sigma(I)$): R_1= 0.1339, wR_2 = 0.3284, R indices (all data): R_1= 0.1860, wR_2 = 0.3948. GOF = 1.189 for 1082 parameters and 329 restraints, largest diff. peak and hole 1.861/–1.120 eÅ^{-3}. CCDC-1894767 contains the supplementary data for this structure.

Solid state analysis

The two dimerization modes show different enclosed cavities due to the inherent flexibility of the hexyl groups along with the halogen bond donors. As shown in Figure S1, the MeCN molecule punctures the surface of the cavity surface, indicating the π···π interaction between the -CN group and phenyl ring of the resorcinarene. The NH···O hydrogen bond between the host and MeOH is also illustrated in the figure.

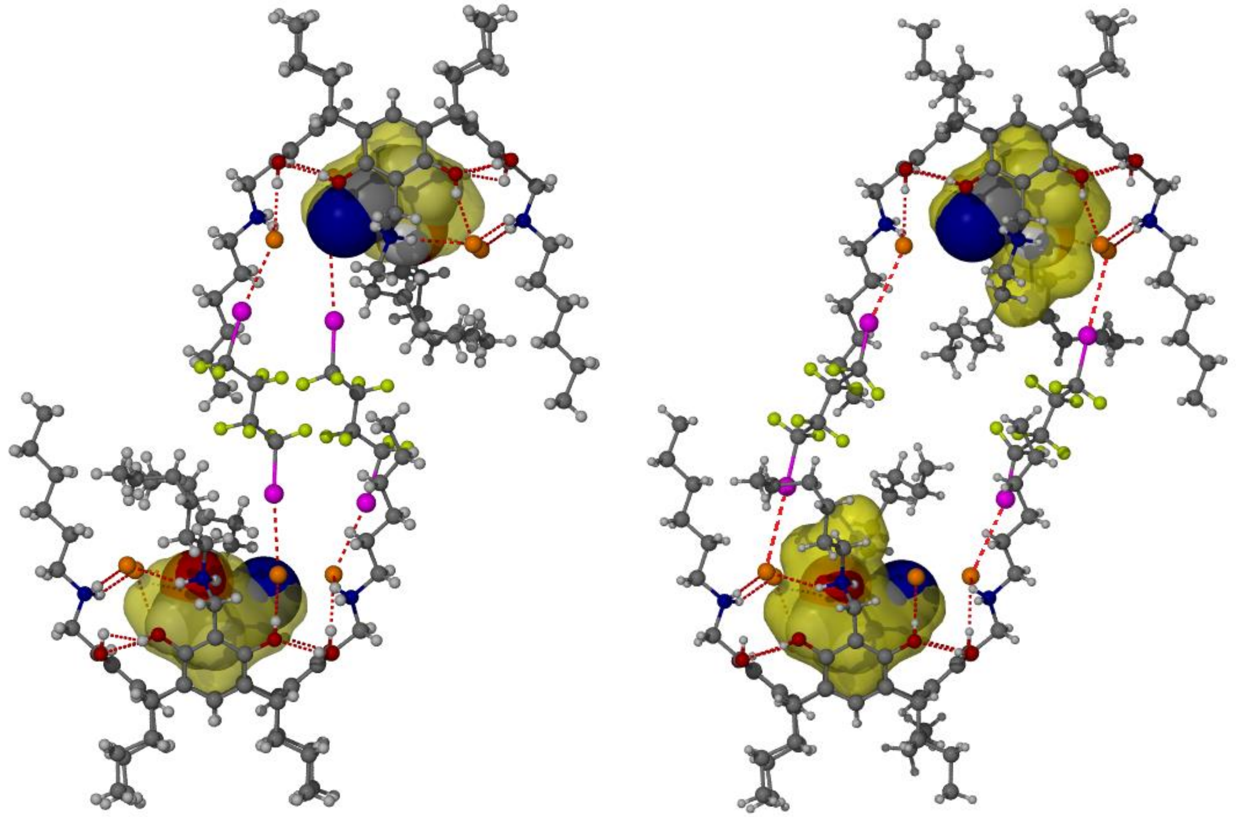

Figure S1. View of the halogen-bonded dimers **1**&DIOFB_A (left) and **1**&DIOFB_B (right), with the enclosed cavity in partial transparent yellow cloud [9]. The included MeOH and MeCN molecules are shown in CPK mode.

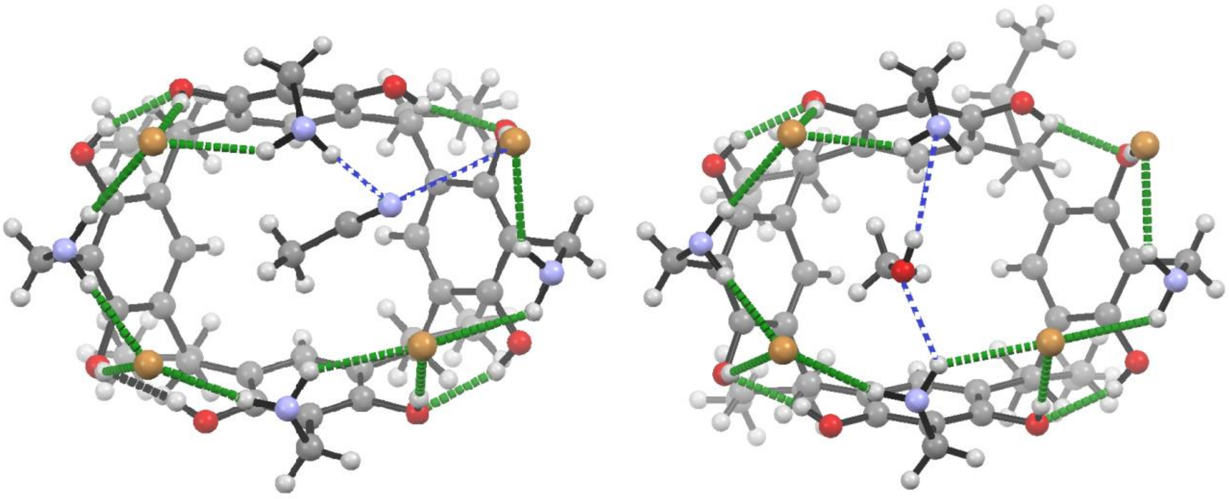

Figure S2. The secondary interactions in the resorcinarene salt (in green) and between the host and guest (in blue). The hexyl groups on the upper rim of the resorcinarene are omitted for clarity. Left: MeCN@**1**; Right: MeOH@**1**.

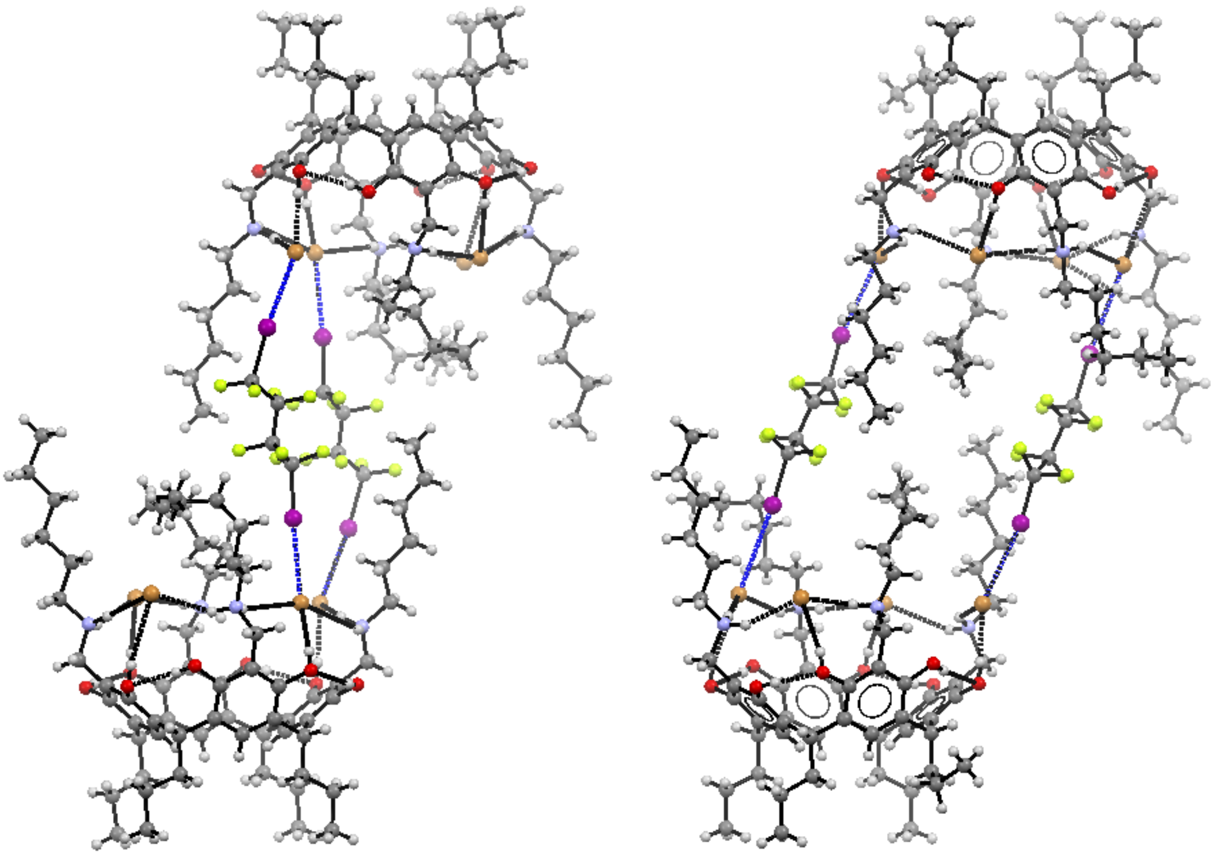

Figure S3. The dimerization modes in **1**&DIOFB_A (left) and **1**&DIOFB_B (right), with halogen bonds as blue broken lines, and the hydrogen bonds as black broken lines. The inclusion guests were omitted for clarity.

III NMR SPECTROSCOPY

For sample preparation, stock solutions of the hex-NARBr receptor (20 mM), the 1,4-diiodooctafluorobutane (DIOFB, 40 mM), and the guests (MeOH, MeCN; 160 mM) were prepared in $CDCl_3$. For the pure hex-NARBr, 250 µL of the stock solution was transferred to an NMR tube and diluted with 312.5 µL of pure $CDCl_3$ providing an 8.8 mM sample concentration. For the pure DIOFB, 125 µL of the stock solution was measured into an NMR tube and diluted with 437.5 µL of pure $CDCl_3$ to give an 8.8 mM sample concentration. For the pure guests, 31.25 µL of the stock solution was transferred into an NMR tube and diluted with 531.25 µL of pure $CDCl_3$ to give an 8.8 mM sample concentration. For a 1:2 (host:DIOFB) mixture, 250 µL of the host, 250 µL of the DIOFB and 62.5 µL of pure $CDCl_3$ provided an 8.8 mM and 17.6 mM sample concentration of the host and DIOFB respectively. For a 1:2:1 (host:DIOFB:guest) mixture, 250 µL of the host, 250 µL of the DIOFB and 62.5 µL of the guest were mixed to give an 8.8 mM, 17.6 mM and 8.8 mM sample concentration of the host, DIOFB and guests respectively.

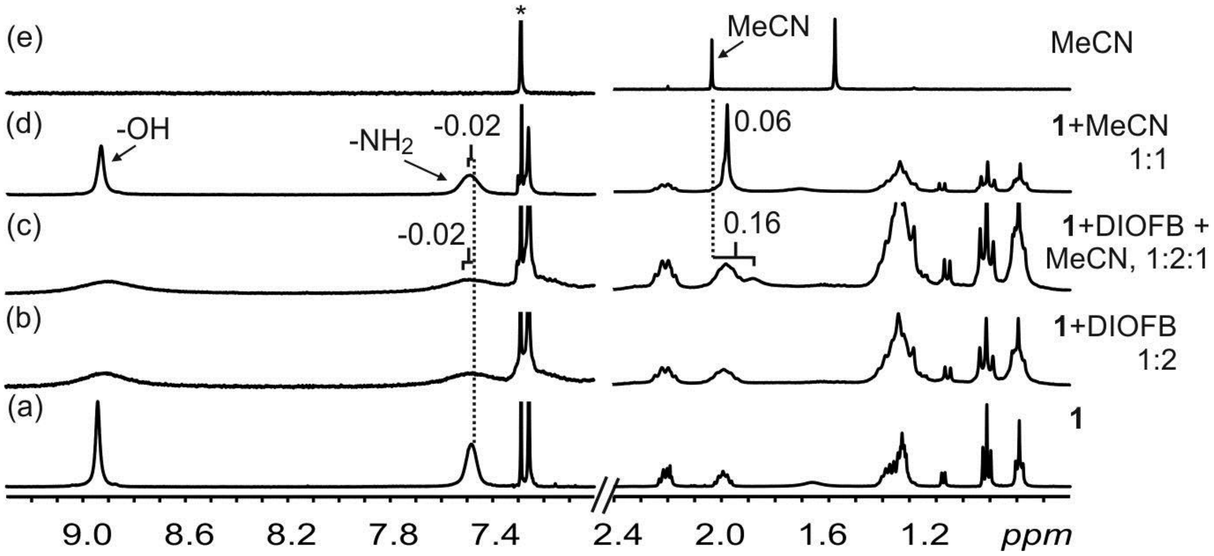

Figure S4. ^{1}H NMR in $CDCl_3$ at 298 K of: a) **1** (10 mM), b) 1:2 mixture of **1** and DIOFB, c) 1:2:1 mixture of **1**, DIOFB and MeCN, d) 1:1 mixture of **1** and MeCN, and e) MeCN (10 mM). The dashed lines give an indication of the signal changes in ppm. The asterisk represents the residual $CHCl_3$ solvent.

IV REFERENCES

1. Beyeh, N. K.; Cetina, M.; Löfman, M.; Luostarinen, M.; Shivanyuk, A.; Rissanen, K. *Supramol. Chem.*, **2010**, *22*, 737–750.
2. Pan, F.; Beyeh, N. K.; Rissanen, K. *J. Am. Chem. Soc.,* **2015**, 137, 10406–10413
3. Airola, K.; Böhmer, V.; Paulus, E. F.; Rissanen, K.; Schmidt, C.; Thondorf, I.; Vogt, W. *Tetrahedron*, **1997**, *53*, 10709–10724.
4. *CrysAlisPro* **2012**, Agilent Technologies. Version 1.171.36.31.
5. Clark, R. C.; Reid, J. S. *Acta Cryst*., *A* **1995***, 51*, 887–897.
6. Sheldrick, G. M. *Acta Cryst., A* **2015**, *71*, 3–8.
7. Dolomanov, O. V.; Bourhis, L. J.; Gildea, R. J.; Howard, J. A. K.; Puschmann, H. *J. Appl. Cryst.,* **2009**, *42*, 339–341.
8. Sheldrick, G. M. *Acta Cryst., C* **2015**, *71*, 3–8.
9. Barbour, L. J. *J. Supramol. Chem.,* **2001**, *1*, 189–191.
